# Importance Modulates the Temporal Features of Self-Referential Processing: An Event-Related Potential Study

**DOI:** 10.3389/fnhum.2017.00470

**Published:** 2017-09-21

**Authors:** Kepeng Xu, Shifeng Li, Deyun Ren, Ruixue Xia, Hong Xue, Aibao Zhou, Yan Xu

**Affiliations:** ^1^Beijing Key Laboratory of Applied Experimental Psychology, National Demonstration Center for Experimental Psychology Education (Beijing Normal University), Faculty of Psychology, Beijing Normal University Beijing, China; ^2^School of Teacher Education, Hexi University Zhangye, China; ^3^School of Psychology, Northwest Normal University Lanzhou, China

**Keywords:** self, social identity, self-referential processing, N200, P200, P300

## Abstract

A growing number of studies have demonstrated preferential processing of self-related information. However, previous research has been limited in examining the distinction between processes related to the self and those related to the non-self, it remains unclear how self-related information with differing levels of importance is processed within the self. The present study examined how the importance of self-related content affects the neural activity involved in self-referential processing. The behavioral results showed that the participants had faster responses to more important self-related content. The event-related potential (ERP) results showed that early attention resources were diverted to the identification of highly important self-related content compared with minimally important self-related content, as reflected by the enhanced P200. Furthermore, the N200 amplitude for highly important self-related content was smaller than for moderately important self-related content which, in turn, were smaller than minimally important self-related content. Moreover, the P300 amplitudes were modulated by the degree of importance of self-related content, whereby a higher importance of self-related content led to larger P300 amplitudes. Taken together, these findings demonstrate an effect of the degree of importance of the self-related content at both behavioral and neurophysiological levels.

## Introduction

The nature of the self represents one of the most important subjects in philosophy and psychology, and has recently attracted attention in neuroscience (Markus and Kunda, 1986; Metzinger and Gallese, 2003; Northoff and Bermpohl, 2004; Northoff et al., 2006; Qin and Northoff, 2011). Several studies have demonstrated that the human brain is equipped with a processing bias toward self-relevant information. For example, behavioral studies showed that the participants’ own names and faces are more rapidly identified (Moray, 1959; Keyes and Brady, 2010) and that information is remembered better when processed in a self-referential encoding than otherwise (Rogers et al., 1977; Kelley et al., 2002). Moreover, a growing number of event-related brain potential (ERP) studies demonstrated preferential processing of self-relevant stimuli. For instance, compared with non-self-related information, self-related information elicited larger P200 amplitudes for names (Chen et al., 2011, 2013), autobiographical information (Hu et al., 2011), and trait adjectives (Mu and Han, 2010; Yu et al., 2010). Furthermore, larger P300 amplitudes were observed for names (Perrin et al., 1999, 2005; Tacikowski and Nowicka, 2010; Zhao et al., 2011), faces (Ninomiya et al., 1998; Scott et al., 2005; Guan et al., 2014, 2015; Kotlewska and Nowicka, 2015), voices (Conde et al., 2015), objects (Miyakoshi et al., 2007), trait adjectives (Yu et al., 2010), hands (Su et al., 2010), possessive pronouns (Zhou et al., 2010), and autobiographical information (Gray et al., 2004; Hu et al., 2011). Additionally, self-related information elicited smaller N200 amplitudes for names (Chen et al., 2013), faces (Sui et al., 2006, 2009; Guan et al., 2014), handwriting (Chen et al., 2008), and possessive pronouns (Zhou et al., 2010) than for non-self-related stimuli. This suggested that the self-referential effect is robust and that self-related information has more priority in capturing the attention resource and involves higher-order cognitive processing at both the early and later stages of information processing.

Generally, these previous studies have investigated the processing of various self-related stimuli. However, they have mainly focused on considering the self-referential processes by assessing differences in the responses to self-related vs. non-self-related stimuli at the behavioral or neural levels. Thus, these studies did not fully consider how self-relevant information with differing levels of importance is processed within the self. The self is a complex structure that includes abundant self-related content, to which is assigned a unique value (Pelham, 1991; Leary, 2004; James, 2013). For example, the individual’s own name (SON) carries a very important significance (Tacikowski et al., 2014). Therefore, the SON elicits a robust electrophysiological P300 response not only during wakefulness (Berlad and Pratt, 1995; Perrin et al., 2005; Zhao et al., 2011; Cygan et al., 2014) but also during sleep or in brain-damaged patients with altered states of consciousness (Perrin et al., 1999; Laureys et al., 2004).

Recently, Tacikowski and Nowicka (2010) compared responses to the self-name and self-face and found that the processing of these two aspects of self-related information did not differ both in reaction times (RTs) and in P300 responses (Tacikowski and Nowicka, 2010). Their results indicated that different types of self-related content, such as the self-name and self-face, activated a similar amount of attentional resources, possibly because the face and the name both have a similar social adaptation value and importance. Additionally, in a functional magnetic resonance imaging (fMRI) study, D’Argembeau et al. (2012) instructed participants to make self-descriptive judgments regarding a variety of trait adjectives during scanning (D’Argembeau et al., 2012). After the scanning, the participants were again presented with the same set of traits and were instructed to rate the level of self-descriptiveness for each trait (i.e., “how important is it for you to possess or not possess this trait?”). Ratings of importance were positively correlated with the medial prefrontal cortex activity, which indicated that the importance of the trait adjective may affect the individual self-referential processing. Therefore, further studies are needed to clarify how the importance of self-related content affects self-referential processing at the electrophysiological level.

Based on these considerations, the present study used the ERP technique, which is known for its high temporal resolution, to investigate the effect of the degree of importance in self-referential processing and its neural correlates. In the present study, we used a typical self-reference effect paradigm in which participants were engaged in judging whether or not a given social identity described them. We chose six representative social identities as stimuli that referred specifically to those aspects of a person that are defined in terms of his or her group memberships. These identities are frequently used in daily life according to a study by Deaux et al. (1995), which included ethnic and religious identities, vocations and avocations, and personal relationships of five identity clusters. Stigmatized identities often represent negative information that may lead to emotional processing. In particular, the Chinese population has relatively few political identities, and most Chinese individuals have no definite political affiliation identity. Therefore, we left out “stigma” and “political affiliation” identity clusters in the present study. Additionally, the self is unique to each person, and the importance of the same social identity as self-relevant content may differ according to the participant. Thus, the participants were queried according to their own standards, and we divided equally six social identities into three categories: highly important self-related content, moderately important self-related content and minimally important self-related content. On the basis of this classification, we analyzed the electrophysiological correlates of self-referential processing using self-related content of different degree of importance. As mentioned above, self-related information elicits larger P200 and P300 amplitudes and smaller N200 amplitude than non-self-related information. We hypothesized that the attentional bias for higher importance of self-related content would be reflected by larger P200 and P300 components, and would also elicit smaller N200 amplitudes.

## Materials and Methods

### Participants

Fifteen healthy students were enrolled in this experiment (9 female participants, mean age 24.6 years, age range 20–27 years). None of the participants had any previous experience with a similar task. All participants were right-handed and had normal or corrected-to-normal visual acuity. Furthermore, all participants gave written informed consent and were paid for their participation. This study was approved by the Ethics Board at the School of Psychology, Beijing Normal University. Written informed consent was obtained from all participants prior to the study, which was approved by the Institutional Review Board of the School of Psychology, Beijing Normal University. The methods were conducted in accordance with approved guidelines.

### Stimuli and Procedure

The basic information and six social identities of the participants were collected 2 weeks prior to the study by using a questionnaire. We adopted six of the participants’ social identities that are frequently used in daily life (ethnicity, nationality, sex, relational roles in family, occupation and age identity) and the corresponding non-self-relevant social identity as stimuli according to Deaux et al. (1995). The experiment consisted of three blocks, each composed of 120 self-relevant stimuli (20 in each of the participants’ social identity, i.e., “
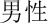
” in Chinese “male”) and 60 non-self-relevant stimuli (10 in each of the other participant’s social identity, i.e., “
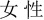
” in Chinese “female”); the sequence of the stimuli was randomized in each block. All stimuli were two- or three-characters Chinese words (i.e., “
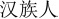
” in Chinese “Han Chinese”, “
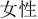
” in Chinese “female”), and were presented visually in a black font on a white background. The size of the stimuli was a minimum of 2.6° × 6°.

During the experiment, the participants were seated in an acoustically and electrically shielded room approximately 85 cm from the screen center. At the beginning of each trial, a small black cross appeared for 300 ms followed by a blank screen, the duration of which randomly varied from 200 ms to 400 ms. Subsequently, a social identity was presented for 1500 ms. The task of the participants was to judge whether or not a given social identity was appropriate to describe the self (yes or no). After the stimulus presentation, a blank screen was presented for 1000 ms. The entire study’s “yes/no” responses were made with the left and right thumbs. Half of the participants were instructed to press the “yes” key with their left thumbs and the “no” key with their right thumbs; the remaining participants responded in the inverse pattern.

After the experiment, the participants were instructed to rate the degree of importance of their self-representation regarding their own social identity, and averagely divided the six social identities into three categories: highly important self-related content, moderately important self-related content, and minimally important self-related content. Six social identities were included in each category (highly, moderately and minimally important) across all the participants. Table 1 summarizes the frequencies of social identities that were included in each category. Chi-square tests revealed that the distribution of the six social identities in each category (highly, moderately and minimally important) did not differ significantly ($\chi_{\left(10\right)}^{2}$ = 15.2, *p* = 0.12), more information in Supplementary Data Sheet S1.

**Table 1:** The frequency of the social identities included in each category across the participants (N [%]).

	N (%)					
	Ethnicity	Nationality	Sex	Family role	Occupation	Age
Highly important	4 (26.7)	4 (26.7)	2 (13.3)	6 (40)	5 (33.3)	9 (60)
Moderately important	2 (13.3)	7 (46.7)	8 (53.3)	5 (33.3)	5 (33.3)	3 (20)
Minimally important	9 (60)	4 (26.7)	5 (33.3)	4 (26.7)	5 (33.3)	3 (20)

### Electrophysiological Recordings

Continuous electroencephalogram (EEG) was recorded from scalp electrodes using the 256-Channel Geodesic Sensor Net (Electrical Geodesics Inc., Eugene, OR, USA). All electrode recordings were initially referenced to vertex (Cz) and re-referenced offline against the average reference. The impedance was kept below 50 KΩ, which is an acceptable setting for this system for its highly impeding amplifiers (Tucker, 1993). Signals were amplified with an online elliptical bandpass filter (0.1–100 Hz) and digitized at a sampling rate of 250 Hz.

During the offline analysis, the data were analyzed using NetStation 4.5.4 analysis software (Electrical Geodesics, Inc., Eugene, OR, USA). For each trial, channels were marked as artifacts if the signal variation exceeded 200 μV; more than 10 channels marked as artifacts were excluded. Trials were excluded if the signal variation of the horizontal and vertical electrooculograms exceeded 140 μV and 55 μV, respectively. The EEG data were digitally filtered via 0.1–30 Hz and re-referenced offline to the average reference for subsequent analysis. The ERPs were segmented to epochs of 1200 ms after stimulus onset with a 200 ms pre-stimulus baseline. After the deletion of the incorrect response trials and artifacts, the ERPs were averaged according to the stimulus type (the degree of importance) into three conditions: highly important, moderately important and minimally important. If the correct and artifact-free trials in any category were less than 30, the participant was then excluded from subsequent analysis. The numbers of trials included in the analysis after the artifacts rejection were ranged from 55 to 109 across all participants, and the number of trials included in highly important, moderately important and minimally important self-related content were comparable (*M* = 80.8/83.1/82.7, respectively).

### Behavioral Data Analysis

Responses were scored as correct if the appropriate key was pressed within a 100–1500 ms period after the stimulus onset. Pressing the wrong key and no responses were treated as incorrect responses. The accuracy rate and mean reaction RTs were separately tested with a repeated-measure analysis of variance (ANOVA) with the stimulus type (highly important, moderately important and minimally important). The analyses were corrected for nonsphericity using the Greenhouse-Geisser correction method when appropriate.

### ERP Analysis

According to the scalp distributions of each ERP component, the mean amplitude of P200 (100–200 ms) and N200 (200–300 ms) were measured and submitted to 3 (stimulus type: highly important, moderately important and minimally important) × 11 (electrode: FC1, FC3, C1, C3, FC2, FC4, C2, C4, FCz, Cz and CPz) two-way repeated measures ANOVAs. The mean amplitudes of P300 (300–700 ms) were measured and submitted to 3 (stimulus type: highly important, moderately important and minimally important) × 15 (electrode: C1, C3, CP1, CP3, P1, P3, C2, C4, Cp2, CP4, P2, P4, Cz, CPz and Pz) two-way repeated measures ANOVAs. The analyses were corrected for nonsphericity using the Greenhouse–Geisser correction method when appropriate. Differences were considered significant at *p* < 0.05, and partial-eta2 ($\eta_{\text{p}}^{2}$) was reported as a measure of the effect size. All statistical analyses were carried out with SPSS (Version 22.0, IBM, SPSS Inc., Chicago, IL, USA).

Electrical source analysis was conducted with GeoSource software^1^ using a low-resolution brain electromagnetic tomography (LORETA) method of constraining the weighted minimum norm inverse solution. Considering P300 as the most robust index for self-referential processing, the source analysis was only implemented during the P300 time-windows.

## Results

### Behavioral Results

Participants recognized their own social identity with a mean accuracy of 98.25% (standard deviation [*SD*] = 1.84; range 94.12%–99.79%), and the non-self-related stimulus with a mean accuracy of 93.51% (*SD* = 7.3; range 79.44%–99.58%). There was a significant main effect of the stimulus type for the degree of importance (*F*_(2,28)_ = 8.56, *p* = 0.08, $\eta_{\text{p}}^{2}$ = 0.38). Pairwise comparisons revealed that the recognition accuracy was higher for highly important self-related content and moderately important self-related content compared with minimally important self-related content (highly important: *t*_(14)_ = 3.02, *p* < 0.01; moderately important:* t*_(14)_ = 2.93, *p* = 0.01); the difference between the highly and moderately important contents was not significant (*t*_(14)_ = 1.10, *p* = 0.29).

The repeated-measures ANOVA of the RTs revealed a significant main effect of the stimulus type (*F*_(2,28)_ = 56.64, *p* < 0.01, $\eta_{\text{p}}^{2}$ = 0.80). The subsequent pairwise comparisons revealed that RTs to highly important self-related content were significantly shorter than RTs to moderately important self-related content (*t*_(14)_ = −3.39, *p* < 0.01), which, in turn, were significantly shorter than minimally important self-related content (*t*_(14)_ = −6.74, *p* < 0.01).

### ERP Results

**Figure 1:**
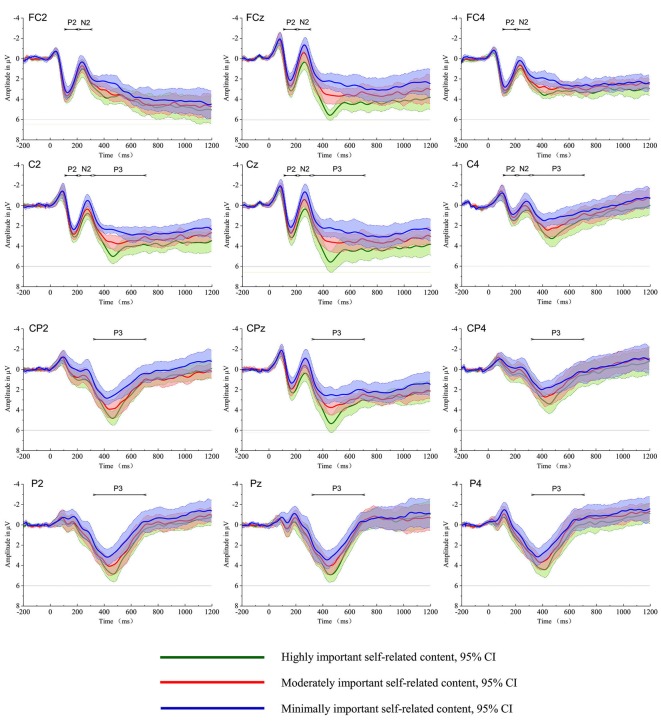
The grand average of the event-related potentials (ERPs) evoked by the highly important, moderately important, and minimally important self-related content conditions at electrodes FC2, FC4, FCz, C2, C4, Cz, Cp2, CP4, CPz, P2, P4 and Pz. The shaded area indicates the 95% confidence interval of the ERPs.

Figure 1 depicts the grand average waveforms for highly important self-related content, moderately important self-related content and minimally important self-related content conditions; the P200, N200 and P300 components were elicited during each of the three conditions.

#### P200 Amplitudes

The repeated measures ANOVA of the P200 amplitude revealed a main effect for the stimulus type (*F*_(2,28)_ = 6.81, *p* < 0.01, $\eta_{\text{p}}^{2}$ = 0.32). The subsequent pairwise comparisons revealed that the P200 amplitude for highly important self-related content was greater than for minimally important self-related content (*t*_(14)_ = 3.81, *p* < 0.01). However, there were no significant differences between highly important and moderately important self-related contents (*t*_(14)_ = 1.95, *p* = 0.07), or between moderately important and minimally important self-related content (*t*_(14)_ = 1.65, *p* = 0.12). The main effect of electrode sites was also significant (*F*_(10,140)_ = 5.87, *p* < 0.01, $\eta_{\text{p}}^{2}$ = 0.29). The neural activity to all types of stimuli was generally greater in FC1 (2.76 μV), FC2 (2.71 μV), FC4 (2.35 μV), and FCz (2.52 μV) than in the other electrodes. There was no interaction effect observed between the stimulus type and electrode (*F*_(20,280)_ = 1.45, *p* = 0.20, $\eta_{\text{p}}^{2}$ = 0.09).

#### N200 Amplitudes

The repeated measures ANOVA revealed a main effect for the stimulus type (*F*_(2,28)_ = 10.17, *p* < 0.01, $\eta_{\text{p}}^{2}$ = 0.42). The subsequent pairwise comparisons revealed that the N200 amplitude for highly important self-related content was significantly smaller than for moderately important self-related content (*t*_(14)_ = 2.35, *p* = 0.03), which, in turn, was significantly smaller than minimally important self-related content (*t*_(14)_ = 2.59, *p* = 0.02). There were no main effects for the electrode sites (*F*_(10,140)_ = 1.66, *p* = 0.18, $\eta_{\text{p}}^{2}$ = 0.10), and no interaction effect between the stimulus type and electrode (*F*_(20,280)_ = 1.77, *p* = 0.11, $\eta_{\text{p}}^{2}$ = 0.11).

#### P300 Amplitudes

The repeated measures ANOVA revealed a main effect for the stimulus type (*F*_(2,28)_ = 18.83, *p* < 0.01, $\eta_{\text{p}}^{2}$ = 0.57). The subsequent pairwise comparisons revealed that the P300 amplitude for highly important self-related content was significantly greater than for moderately important self-related content (*t*_(14)_ = 3.98, *p* < 0.01), which, in turn, was significantly greater than minimally important self-related content (*t*_(14)_ = 2.21, *p* = 0.04). The main effect of the electrode sites was also significant (*F*_(14,196)_ = 7.02, *p* < 0.01, $\eta_{\text{p}}^{2}$ = 0.33). The neural activity to all types of stimuli was generally greater in C1 (1.54 μV), C2 (2.01 μV), and Cz (1.64 μV) than in the other electrodes. There was no interaction effect between the stimulus type and electrode (*F*_(28,392)_ = 1.55, *p* = 0.21, $\eta_{\text{p}}^{2}$ = 0.1).

The estimated source regions contributing to the P300 divergence between ERPs elicited by the three stimulus types (highly, moderately, and minimally important) during 300–700 ms are illustrated in Table 2. In particular, the results revealed a significant effect of the stimulus type in the medial frontal gyrus (*F*_(2,28)_ = 4.66, *p* = 0.03, $\eta_{\text{p}}^{2}$ = 0.25), precuneus (*F*_(2,28)_ = 6.42, *p* = 0.02, $\eta_{\text{p}}^{2}$ = 0.31), orbital gyrus (*F*_(2,28)_ = 4.92, *p* = 0.03, $\eta_{\text{p}}^{2}$ = 0.26), and right superior parietal lobule (*F*_(2,28)_ = 10.72, *p* < 0.01, $\eta_{\text{p}}^{2}$ = 0.43). The subsequent pairwise comparisons revealed that the activity in the medial frontal gyrus was greater for the highly important self-related content and moderately important self-related content compared with the minimally important self-related content (*t*_(14)_ = 2.81, *p* = 0.01; *t*_(14)_ = 2.43, *p* = 0.02). However, the difference between the highly and moderately important contents was not significant (*t*_(14)_ = 1.40, *p* = 0.18). The activity in the precuneus for highly important self-related content was significantly greater than that for moderately important self-related content (*t*_(14)_ = 2.13, *p* = 0.05), which, in turn, was significantly greater than minimally important self-related content (*t*_(14)_ = 2.96, *p* = 0.01). The activity in the orbital gyrus was greater for highly important self-related content and moderately important self-related content compared with that for minimally important self-related content (*t*_(14)_ = 2.97, *p* = 0.01; *t*_(14)_ = 2.40, *p* = 0.03). However, the difference between the highly and moderately important contents was not significant (*t*_(14)_ = 1.43, *p* = 0.17). The activity in right superior parietal lobule for highly important self-related content was significantly greater than for moderately important self-related content (*t*_(14)_ = 2.36, *p* = 0.03), which, in turn, was significantly greater than minimally important self-related content (*t*_(14)_ = 3.20, *p* < 0.01).

**Table 2:** Detailed low-resolution brain electromagnetic tomography (LORETA) results of the high time resolution analysis with significant differences in brain electrical activity during three conditions from 300 ms to 700 ms.

Hemisphere	Cerebral region	Brodmann areas	*xyz*-coordinates	*F*	Sig.
Medial	Medial frontal gyrus	11	4, 52, −13	4.66	0.03
	Precuneus	7	4, −60, 43	6.44	0.02
	Orbital gyrus	11	4, 52, −20	4.92	0.03
Right	Superior parietal lobule	39	53, −60, 29	10.72	<0.01

## Discussion

The present study examined how the importance of information modulates the neural activity of self-related content in self-referential processing. To this end, we recorded ERPs elicited by self-describing judgments for three categories of social identities with varying degrees of importance to the participants. The behavioral results showed that the participants were more accurate in recognizing highly important self-related content and moderately important self-related content than minimally important self-related content. Furthermore, the participants made faster responses to highly important self-related content than to moderately important self-related content and faster responses to moderately important self-related content than to minimally important self-related content. The ERP results provided more information on the underlying process of this response bias. First, more early attention resources were diverted to the identification of highly important self-related information than minimally important self-related information, as reflected by the enhanced P200 amplitude. Second, the N200 amplitude for highly important self-related content was smaller than for moderately important self-related content, and the N200 amplitude for moderately important self-related content was smaller than for minimally important self-related content. Finally, the higher importance of self-related content led to deeper processing during the evaluative processing stages, as reflected by the larger P300 amplitude.

Automatic processes were indexed according to the P200 component, which is considered to reflect the ongoing automatic monitoring of semantic meaning and significance of incoming information (Crowley and Colrain, 2004; Shestyuk and Deldin, 2010). Moreover, the P2 component was speculated to represent an index of attention responses to highly arousing and highly attention-grabbing stimuli (Mu and Han, 2010; Liu et al., 2013; Tacikowski et al., 2014). Additionally, a growing number of brain ERP studies demonstrated that self-related information elicits larger P200 amplitudes compared with non-self-related information (Yu et al., 2010; Chen et al., 2011, 2013). Thus, the P200 amplitude for highly important self-related content was greater than that for minimally important self-related content in the present study. This indicated that highly important self-related information could be more arousing and attention-capturing for some participants than for others. However, highly important self-relevant and moderately important self-relevant information, or moderately important self-relevant and minimally important self-relevant content were not significant for this component, most likely because there are no prominent differences between each pair of self-relevant contents. Therefore, the degree effect for moderately important self-relevant and minimally important self-relevant content may occur at later processing stages.

During the 200–300-ms time interval, an obvious fronto-central N200 component was observed in each of the three experimental conditions. The N200 amplitude for highly important self-related content was smaller than that for minimally important self-related content. The N200 is thought to index early higher-order operations related to the discrimination and categorization of stimuli (Patel and Azzam, 2004). Thus, this component may represent a frontier between automatic and controlled processing phases (Carretié et al., 2004; Li et al., 2008). As such, the decreased N200 amplitude observed for the self-related content of increased importance may indicate that more important self-related content is more easily retrieved. Similarly, previous studies demonstrated that smaller N200 amplitudes were elicited by highly self-relevant stimuli than by less self-relevant stimuli (Chen et al., 2011), and smaller N200 amplitudes were elicited by individually self-relevant stimuli than by collectively self-relevant stimuli (Chen et al., 2013). These findings suggested that some self-related content (based on its important adaptive value to the individual) can be retrieved more easily and with less top-down cognitive resource consumption. Therefore, in this study, we hypothesized that the N200 amplitude may reflect the initial identification of the importance of self-related content in the early stages of self-descriptive judgments.

The P300 component was previously established as a valid index for self-referential processing (Knyazev, 2013). As expected, a clear P300 component was elicited by all three experimental conditions. The maximum was over central-parietal scalp sites, and larger P300 amplitudes were elicited by highly important self-related content than by moderately important self-related content. The P300 amplitudes for this latter, in turn, were significantly greater than for the minimally important self-related content. P300 is known to reflect the engagement of higher-order cognitive functions (Farwell and Donchin, 1991; Patel and Azzam, 2004; Miyakoshi et al., 2007). These higher-order cognitive functions include context updating, evaluation of the stimuli, and allocation of the attentional resources and associative memory processes (Polich, 2007). We used a simple discrimination task in the present study. Thus, the functional meaning of the P300 component most likely reflects the cognitive evaluation of stimulus significance in addition to the mobilization of higher-order attentional resources to a task-relevant target event (Polich, 2007; Conde et al., 2015). Therefore, self-related content eliciting larger P300 amplitudes for higher degrees of importance should be due to their differential importance. More importantly, studies on the neural mechanisms of self-relevant processing have indicated that P300 is also an index of attention and cognitive evaluation to self-related stimuli (Su et al., 2010; Tacikowski and Nowicka, 2010; Conde et al., 2015). Furthermore, a larger P300 elicited by self-related stimuli was attributed to the meaningfulness of self-relevant information (Johnson, 1986). It is thus conceivable that the larger P300 amplitudes to more important self-related information reflected an increased allocation of attention, as well as a more elaborate and deeper processing.

The electrical source analysis revealed that the activity in the medial frontal gyrus, precuneus, orbital gyrus and right superior parietal lobule was modulated by the degree of importance of the self-related content. Considering that those regions are associated with self-referential processing (Northoff et al., 2006; Uddin et al., 2007), the degree of activity thus represents the depth of the self-referential processing. Moreover, our results were congruent with a previous study that reported that the ratings of personal importance were positively correlated with the activity in a region of the medial prefrontal cortex, precuneus, and inferior parietal lobe (D’Argembeau et al., 2012). Thus, the present findings provided support for the view that the importance of the self-related content modulated the processes that evaluate, select, and organize the mental representations on the basis of their personal relevance.

According to the self-categorization theory, the self can be classified into the individual self and the collective self (Brewer, 1991; Brewer and Gardner, 1996). The collective self refers to the cognition of group memberships, relationships, and social roles (e.g., daughter, teammate and nationality), while the individual self involves the cognition related to personal traits, states, or behaviors (e.g., kind, smart and optimistic). One limitation of the present study was that we compared the social identities only at a collective level. To understand further how the importance affects the processing of self-related information, the content of the individual self should be addressed in future work (e.g., autobiographical information).

In summary, we found an effect of the degree of importance of the self at both the behavioral and neurophysiologic levels when self-related content was processed. Stimuli differing in the extent of importance are processed differently in the early attentional and late evaluation cognitive processing stages, as reflected by the RTs, and the P200, N200 and P300 potentials. More important self-related information captured attention more quickly at early time-points and received more elaborate and deeper processing in the late cognitive evaluation stages. Taken together, these findings indicated that a processing advantage of self-related information is present in differentiating between the self and non-self and in differential importance variations. Further research could extend this effect to self-related stimuli belonging to other categories.

## Author Contributions

KX and SL designed the study. DR, RX and HX performed the data collection. KX and SL analyzed the data. KX, SL, AZ and YX wrote the manuscript. All authors participated in revising the manuscript.

## Conflict of Interest Statement

The authors declare that the research was conducted in the absence of any commercial or financial relationships that could be construed as a potential conflict of interest.

## Footnotes

^1^http://www.egi.com

## Supplementary Material

Supplementary Data Sheet S1 Dataset of this paper is available online at: https://figshare.com/s/a90593f4c6e5742230fd
